# Antimicrobial Resistance Profile of Zoonotic Clinically Relevant WHO Priority Pathogens

**DOI:** 10.3390/pathogens13111006

**Published:** 2024-11-15

**Authors:** Elaine Meade, Mark Anthony Slattery, Mary Garvey

**Affiliations:** 1 Department of Life Science, Atlantic Technological University, F91 YW50 Sligo, Ireland; elaine.meade@atu.ie; 2 Veterinary Practice, Manorhamilton, F91 DP62 Leitrim, Ireland; 3 Centre for Precision Engineering, Materials and Manufacturing Research (PEM), Atlantic Technological University, F91 YW50 Sligo, Ireland

**Keywords:** ESKAPE, critical, fungal, resistance, mortality, zoonosis

## Abstract

The World Health Organization announced critically important bacterial and fungal pathogens displaying alarming levels of antimicrobial resistance, which currently represent difficult-to-treat cases of morbidity. Within this grouping, the ESKAPE pathogens (*Enterococcus faecium*, *Staphylococcus aureus*, *Klebsiella pneumoniae*, *Acinetobacter baumannii*, *Pseudomonas aeruginosa*, and *Enterobacter* species) are causative of significant morbidity and mortality. Studies described herein demonstrate the presence of critically important fungal and ESKAPE bacterial species in companion animals which are zoonotic in nature. The relationship between the environment, animals, and human infectious disease has long been recognized as part of One Health. This research investigates the resistance patterns of isolated zoonotic pathogens using recognized in vitro methodologies, namely disk diffusion, minimum inhibitory concentration testing, and genetic screening. Antibiotic susceptibility testing and gene analysis demonstrated an association between multi-drug resistance and extended beta spectrum lactamase production in critical-priority bacteria. *Escherichia coli*, *Klebsiella pneumoniae*, *Acinetobacter baumannii*, and *Pseudomonas aeruginosa* exhibit great levels of multi-drug resistance. Fungal isolates demonstrated high levels of resistance, with Amphotericin B proving the most effective antifungal agent investigated. The level of antimicrobial resistance present in clinically relevant bacterial and fungal pathogens isolated from animal cases of morbidity in this study is alarming. In conclusion, this study shows that animals can act as a reservoir facilitating the transmission of antibiotic-resistant pathogens and genes zoonotically.

## 1. Introduction

The proliferation of antimicrobial resistance (AMR) causes alarming rates of morbidity and mortality, globally. The emergence and re-emergence of pathogenic species, zoonotic transmission, and treatment-resistant infectious disease are major public health challenges. The World Health Organization (WHO) announced a fungal priority pathogen list in October 2022 specifying fungal pathogens of major concern to public health safety [1]. *Cryptococcus neoformans* and *Candida ablicans* are listed as critically important fungal pathogens with *Candida parapsilosis*, *Candida tropicalis*, and *Nakaseomyces glabrata* (*Candida glabrata*) listed as high importance [1]. This fungal pathogen list is a companion of the bacterial pathogen list announced in 2017 detailing bacterial pathogens of critical, high, and medium importance and last-resort antibiotics. The bacterial priority pathogen list contains the ESKAPE bacterial species, namely, *Enterococcus faecium*, *Staphylococcus aureus*, *K. pneumoniae*, *A. baumannii*, *P. aeruginosa*, and *Enterobacter* spp., amongst other important species [2]. These ESKAPE bacteria cause incidence of morbidity and mortality, with AMR and extensively drug-resistant (XDR) pathogens leading to increased economic impacts and burdens on healthcare facilities [3]. For example, small-colony variants of *S. aureus* can cause persistent resistant dermal infections whereas *S. aureus* bacteraemia represents a ca. 30% mortality rate in patients [4]. The relationship between the environment, animals, and human infectious disease has long been recognized as part of One Health. These fungal species and ESKAPE bacterial species are zoonotic in nature with reverse zoonosis (anthropozoonosis) of AMR species such as methicillin-resistant *Staphylococcus aureus* (MRSA) also a factor in disease transmission [5]. Domestic and therapy animals are recognized as routes of transmission of many bacterial pathogens including *Enterococcus* spp., *Escherichia coli*, *K. pneumoniae*, MRSA, vancomycin-resistant *enterococci* (VRE), *A. baumannii*, *P. aeruginosa* [6] and fungal pathogens including *Candida species* and *Cryptococcus neoformans* [2] having significant multidrug-resistance (MDR) profiles. The extensive application of antibiotics in animals has proliferated the transmission and emergence of MDR pathogens of clinical significance. The transmission of AMR via mobile genetic elements and plasmids has promoted the emergence of resistance phenotypes [7], which disseminate in the environment and animal hosts. AMR is clinically evident by the need for increased concentrations of antimicrobial agents to achieve a minimum inhibitory concentration (MIC) of the pathogenic species. Antibiotic tolerance resulting in treatment failure has also emerged as a public health risk, where bacterial species survive high doses of bactericidal antibiotics and establish persister cells post antibiotic exposure [8,9]. The European Committee on Antimicrobial Susceptibility Testing (EUCAST) publishes tables annually on clinical breakpoints, Clinical and Laboratory Standards Institute (CLSI) breakpoints, detailing concentrations of antimicrobials against pathogenic species allowing for categorization as susceptible (S), intermediate (I), and resistant (R) for disk diffusion susceptibility testing and MICs [10]. As part of antimicrobial stewardship, proper detection and AMR profiling of zoonotic-resistant species is required in companion animals. This study identifies many WHO-priority pathogens in animal cases of morbidity that may transmit zoonotically to humans. Extensive drug resistance is demonstrated in bacterial and fungal species, supporting the need for a comprehensive One Health approach to mitigate AMR and safeguard human health. Resistance profiles are determined for zoonotic WHO priority fungal pathogens and priority ESKAPE pathogens isolated from animal infectious disease utilizing the EUCAST susceptibility testing protocols. Increasingly, studies investigate the application of alternative biocontrol options, including antimicrobial peptides (AMPs), as prophylactic and metaphylactic antimicrobial agents in food-producing animals [11]. The antimicrobial activity of two AMPs against isolated MDR species is also determined to determine their efficacy as metaphylactic agents.

## 2. Materials and Methods

### 2.1. Animal Morbidity Relating to Infection

Bacterial and fungal isolates were obtained from diagnostic testing of companion animals (feline and canine) manifesting with morbidities including bacteraemia, renal infection, oral infection, dermal irritation and infection, cervical infection, abortion, and fertility issues. Numerous cases entered the veterinary clinic displaying symptoms that indicated microbial disease requiring culture and identification of causative pathogens. The veterinary clinic is located in the Northwest region, Republic of Ireland. Samples were obtained via swabbing using sterile swabs for oral infections and cervical infections, blood samples were cultured in suspected cases of bacteremia, urine samples in the case of suspected renal infection, and milk samples in the case of suspected intramammary infections. Morphological, biochemical, and molecular assays, namely polymerase chain reaction (PCR) assays, were then utilized to identify species present as per Section 2.2. Antimicrobial susceptibility testing was performed on all isolates to determine the best treatment options, particularly in cases of chronic infection and comorbidities as per Section 2.4. Pathogenic species isolated from the cases and used for this study are as follows: VRE, MRSA, *Klebsiella pneumoniae*, *Pseudomonas aeruginosa*, *Acinetobacter baumannii*, *Enterobacter asburiae* and *Escherichia coli* (bacterial), and *Candida albicans*, *Candida glabrata*, *Candida tropicalis*, *Candida krusei*, *Candida parapsilosis*, and *Cryptococcus neoformans* (fungal). All species were identified via microbial, biochemical, chromogenic, and PCR methods as described in Section 2.2 and Section 2.3.

### 2.2. Diagnostic Microbial Isolation, Identification, Culture, and Maintenance

**Figure 1:**
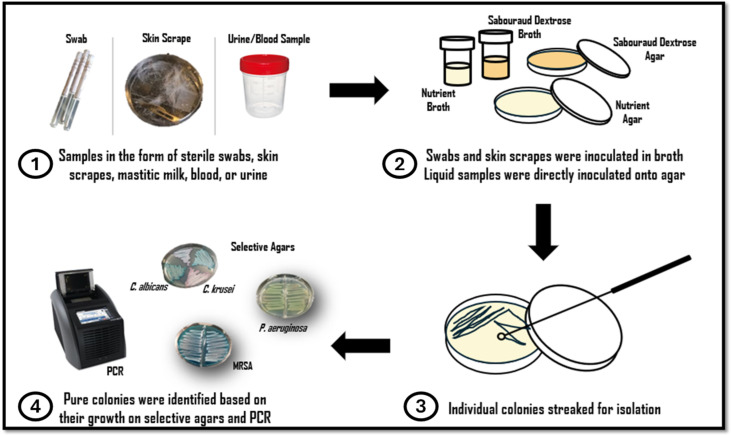
Schematic diagram detailing (1) how samples of infection were collected and received, (2) how samples were cultured, (3) how colonies were isolated, and (4) how isolates were identified based on growth on selective agars and PCR.

Registered clinical personnel at the veterinary clinic provided clinical samples in the form of sterile swabs, skin scrapes, mastitic milk, blood, or urine samples. Swabs and skin scrapes were inoculated in sabouraud dextrose broth/nutrient broth and incubated on an orbital shaker at 120 rpm at varying temperatures (25 °C, 30 °C and 37 °C) for up to 72 h. Samples were then streaked onto fresh sabouraud dextrose agar/nutrient agar plates, and individual colonies were picked and streaked for isolation. Liquid samples (blood, urine) were directly inoculated onto sabouraud dextrose broth/nutrient agar, and individual colonies were streaked for isolation. Colony morphology on relevant agars was observed. Gram staining was performed on isolated species, and biochemical testing was conducted, e.g., catalase production, oxidase test, indole test, MRVP test, mobility test, and Triple Sugar Iron Agar test (TSIA). Isolated colonies were also identified based on their growth on selective agars and PCR as per Section 2.3 (Refer to Figure 1 for a schematic diagram detailing how samples were collected, cultured, isolated, and identified based on growth on selective agars and PCR).

### 2.3. PCR Identification

For all test isolates, the direct colony PCR method was performed. Briefly, a single colony of the test isolate was picked from a fresh culture plate using a sterile micropipette tip and suspended in a 1.5 mL Eppendorf tube containing 100 µL sterile deionized water. The Eppendorf containing the suspended colony was then heated on a heat block at 94 °C for 10 min. Following the incubation period, the Eppendorf was centrifuged at 12,000 rpm for 10 min. A master mix containing 17 µL Red Taq DNA Polymerase 1.1X Master Mix 1.5 mM MgCl_2_ (VWR, Dublin, Ireland), 0.5 µM of selected forward primer (ITS1-F or ITS_8F), and 0.5 µM of selected reverse primer (ITS4 or ITS_U1492R) was prepared. Refer to Table 1 for full primer details. To this master mix, 1 µL of the supernatant of the previously centrifuged Eppendorf containing the suspended colony was added, totaling a final PCR reaction volume of 20 µL. DNA amplification was performed in a thermocycler (VWR, Dublin, Ireland) using the recommended parameters. Following DNA amplification, the PCR products were examined on a 1% *w*/*v* agarose gel run at 110 volts for 45 min. Successful reactions were sent to GATC (Eurofins Genomics, München, Germany) for cleanup and gene sequencing of products.

### 2.4. Antimicrobial Resistance Profile of Zoonotic Isolates

Antimicrobial-susceptibility patterns of isolated strains were assessed using the disk diffusion (Kirby–Bauer) method and the gradient concentration strip (MIC) method. A range of antibiotics from varying drug classes were used in the susceptibility testing of bacterial isolates, including erythromycin (E), azithromycin (AZM), vancomycin (VA), dalbavancin (DAL), quinupristin/dalfopristin (QDA), (amoxicillin (AML), amoxicillin-clavulanic acid (AMC), piperacillin (PIP), piperacillin-tazobactam (TZP), cefixime (CFM), ceftazidime (CAZ), ceftriaxone (CRO), cefepime (FEP), cefiderocol (FDC), doripenem (DOR), meropenem (MEM), aztreonam (ATM), aztreonam-avibactam (AZA), ciprofloxacin (CIP), gentamicin (CN), doxycycline (DXT), colistin (CS), chloramphenicol (C), trimethoprim (TM), and trimethoprim-sulfamethoxazole (1:19) (SXT). To note, gram-negative bacterial isolates were screened for resistance to colistin only, where EUCAST recommend the Broth Disk Elution MIC Method to confirm colistin resistance in isolates.

Antifungal agents tested included Amphotericin B (AMP B), fluconazole, caspofungin, and flucytosine. For all bacterial and fungal isolates, the antimicrobial activity of two AMPs, including Bacitracin (BA) and Daptomycin (DAP), was also assessed using the gradient concentration strip (MIC) method. All antimicrobial susceptibility disks and MIC strips were sourced from Launch Diagnostics, Sligo, Ireland.

#### 2.4.1. Disk Diffusion Assay

The disk diffusion assay was performed for all test isolates using a range of antimicrobial impregnated disks, where antimicrobial agents tested are listed in Section 2.4, ‘Antimicrobial Resistance Profile of Zoonotic Isolates’. Microbial suspensions were prepared by picking a single colony of the respective test cultures previously grown on Muller–Hinton or sabouraud dextrose agar and suspending it in sterile PBS. The microbial cell densities were then adjusted to 1 × 10^8^ CFU/mL using sterile PBS. The resultant microbial inoculums were streaked onto Muller–Hinton or sabouraud dextrose agar using a sterile cotton swab, ensuring to cover the entire surface of the plate with the suspension. Antimicrobial impregnated disks (Scientific Laboratory Supplies, Dublin, Ireland) of each respective antimicrobial agent were placed on the inoculated plates using a disk dispenser (Fisher Scientific, Dublin, Ireland). Muller–Hinton plates were then inverted and incubated for 24 h at 37 °C. Sabouraud dextrose plates were inverted and incubated for 24 h at 30 °C for all fungal species, with the exception of the *C. neoformans* isolate, which was incubated for 48 h at 30 °C. Zones of inhibition were measured in millimeters, and antibiotic susceptibility results were interpreted as resistant (R), susceptible (S), or susceptible increased exposure (I) according to the EUCAST guidelines.

#### 2.4.2. MIC-Gradient Test Strip Assay

MICs for all bacterial and fungal isolates were determined by the gradient concentration strip method (Liofilchem MTS, Launch Diagnostics, Ireland), whereas a predefined exponential gradient of the antimicrobial drug was impregnated on a paper strip across 15 twofold dilutions.

Bacterial and fungal inoculums were prepared by suspending a single colony of the respective test species in sterile PBS and adjusting to a final cell density of ca. 1 × 10^8^ CFU/mL. For bacterial species Muller–Hinton agar plates were used, while for fungal species RPMI 1640 (supplemented with MOPS 0.165 M and 2% glucose) (Launch Diagnostics, Ireland) agar plates were used. Sabourad dextrose agar plates were used for *C. neoformans* isolate, whereas RPMI 1640 medium is not recommended for *C. neoformans* growth. Like previously described, a microbial lawn of the respective test isolate was streaked across the surface of the respective agar plate using a sterile cotton swab, ensuring an even distribution of the inoculum. A test strip of the respective antimicrobial agent was then applied to the surface of the agar. Plates were inverted and incubated for 24 h at 37 °C for bacterial species. Fungal species were incubated at 30 °C for 24 h, except for *C. neoformans*, which was incubated for 48 h at 30 °C. After the incubation period (and only if an even lawn of growth was distinctly visible), MICs were read visually where the inhibition ellipse intersects the MIC scale of the strip. MICs for each antimicrobial agent were interpreted according to the manufacturer’s instructions, where MICs for bactericidal drugs were read at the point of complete inhibition of all growth, i.e., haze, macrocolonies/microcolonies, or isolated colonies within 3 mm from the strip were read as growth. For bacteriostatic drugs, MICs were read at 80% inhibition of growth when trailing endpoints were seen. Results were then compared to standardized EUCAST clinical breakpoints, where species were classed as resistant (R), susceptible (S), or susceptible, increased exposure (I). MIC test strips containing bacitracin and daptomycin were also tested in the same manner as described for the antimicrobial MIC test strips.

### 2.5. Phenotypic Detection of ESBL and AmpC-Producing Bacterial Isolates

Gram-negative Enterobacterale isolates, including *E. coli*, *K. pneumoniae*, and *E. asburiae*, were phenotypically screened for the presence of *ESBL* genes by inoculation on CHROMagar ESBL selective (CHROMagar, Paris, France) and Double Disk Synergy, while the presence of *AmpC* genes was screened for using combination test strips.

**Figure 2:**
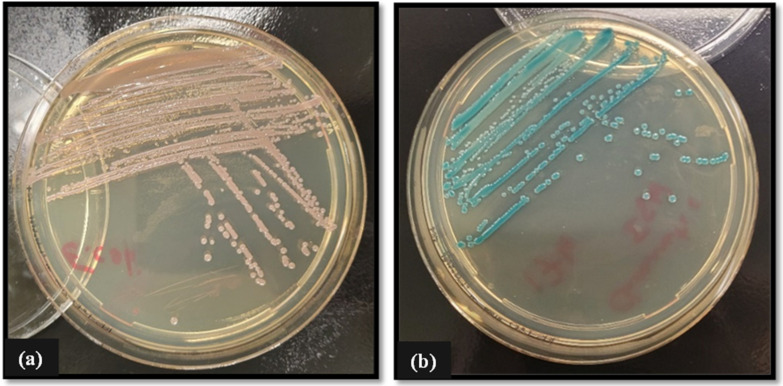
Growth of ESBL-producing (**a**) *E. coli* (pink-reddish-mauve colonies indicating a positive result) and (**b**) *K. pneumonia* (green-blue colonies indicating a positive result) isolates post 24 h incubation at 37 °C on ‘Chromatic ESBL’ chromogenic media for determination of ESBLs in Enterobacterale species. Reading of results for chromogenic agar is followed as per manufacture guidelines as provided on the manufacturer’s website. See link CHROMagar™ ESBL-Chromagar (https://www.chromagar.com/en/product/chromagar-esbl/, accessed on 7 November 2024).

**Figure 3:**
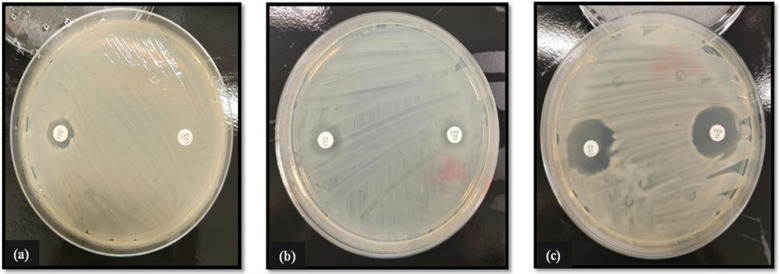
Double Disk Synergy test for phenotypic analysis of ESBL activity in Gram-negative Enterobacterale isolates using cefpodoxime (CPD) and cefpodoxime + clavulanic acid (CD) disks. ESBL production was judged if the zone diameter ratios for the antibiotic comparative to antibiotic plus inhibitor was ≥5 mm. Imaged (**a**) *E. coli* (testing positive for ESBL production), (**b**) *K. pneumoniae* (testing positive for ESBL production), and (**c**) *A. asburiae* (testing negative for ESBL production). Reading of results for Double Disk Synergy testing is followed as per manufacturer guidelines.

All Enterobacterale isolates were first screened for the presence of *ESBL* genes using CHROMagar ESBL selective. Briefly, a single colony was picked using a sterile inoculating loop and streaked onto the surface of freshly prepared CHROMagar ESBL plates. Plates were then inverted and incubated for 24 h at 37 °C, and results were interpreted according to the manufacturer’s instructions (CHROMagar, Paris, France) (see Figure 2). For the Double Disk Synergy test, combination pairs of antibiotic discs containing cefpodoxime (10 µg/disc) alone and cefpodoxime in combination with clavulanate (10:1 µg/disc) were used. Briefly, a bacterial suspension was prepared by seeding a single colony picked from a freshly grown culture in sterile PBS. The resultant inoculum was adjusted to a cell density of 1 × 10^8^ CFU/mL using sterile PBS. Subsequently, a sterile swab was introduced to the suspension and then streaked across the surface of a Muller–Hinton plate, ensuring even distribution to create a bacterial lawn. The combination disks were then placed at either end of the plate using a disk dispenser, and the plate was inverted and incubated at 37 °C for 24 h. Isolates were deemed positive for ESBL production when the zone diameter produced by the cefpodoxime/clavulanate disk was 5 mm greater than that of the cephalosporin disc alone (See Figure 3).

**Figure 4:**
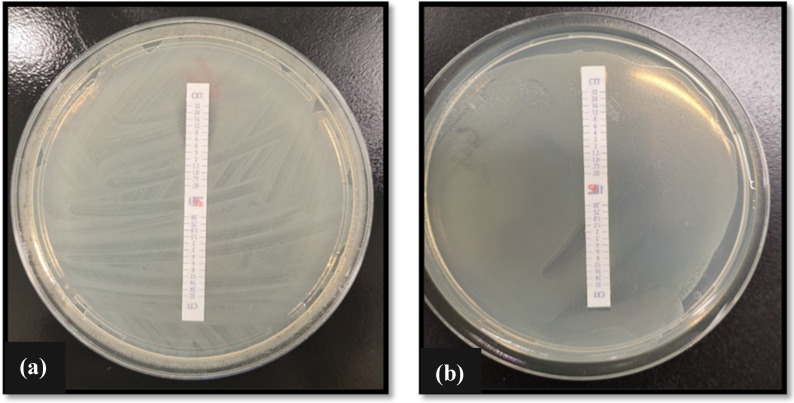
Phenotypic confirmation of *AmpC* production in Enterobacterale isolates (**a**) *K. pneumoniae* and (**b**) *E. coli* using MIC combination test strips containing cefotetan (CTT)/cefotetan + cloxacillin (CXT). *AmpC* production was assumed if the MIC ratios of antibiotic comparative to antibiotic plus inhibitor was ≥8.

Combination test strips containing cefotetan/CTT (0.5–32 μg/mL) and cefotetan (0.5–32 μg/mL) plus cloxacillin/CXT were used to screen for the presence of *AmpC* genes in Enterobacterale species. Briefly, a bacterial inoculum of the respective test species was prepared as previously described. Subsequently, a bacterial swab was introduced to the respective suspension, and a bacterial lawn was streaked across the surface of a Muller–Hinton agar. The combination test strip was then placed on the center of the plate, and the plate was inverted and incubated at 37 °C for 24 h. MICs were read at both ends of the strip at the point of intersection between the inhibition ellipse and the MIC scale of the strip. The presence of *AmpC* enzymes was indicated if the MIC ratios for the antibiotic alone compared with the antibiotic plus inhibitor was ≥ 8 (i.e., if the ratio CTT/CXT was ≥ 8) or by the appearance of a phantom zone or deformation of the CTT ellipse (See Figure 4). Confirmation of β-lactamase genes was determined using PCR (see the preceding Section 2.6, ‘Genotypic Detection of ESBL and AmpC-Producing Isolates’.

### 2.6. Genotypic Detection of ESBL and AmpC-Producing Isolates

All isolates found to be phenotypically positive for β-lactamases were further assessed for the presence of *blaTEM*, *blaSHV*, *blaOXA*, *blaCTX*, *blaPER*, *and blaAmpC* genes by PCR assay using specific primers as per Meade et al. (2021) [12]. Refer to Table 1 for primer information. Standard strains of *E. coli* (ATCC BAA-201) and *K. pneumoniae* (ATCC 700603) producing ESBL and *AmpC* enzymes were used as positive controls, while *E. coli* (ATCC 25922) was used as a negative control. Bacterial DNA of selected isolates was extracted using the boiling method. Briefly, an overnight-grown colony of each bacterial test species was suspended in an Eppendorf containing 1 mL of sterile distilled water and placed in a heat block, previously set to a temperature of 94 °C, for 10 min. The suspensions were subsequently centrifuged for 5 min at 13,000 rpm. PCR was performed in a total reaction volume of 20 µL containing 17 µL Red Taq DNA Polymerase 1.1X Master Mix 1.5 mM MgCl_2_ (VWR, Dublin, Ireland), 0.5 µM of each selected primer pair and 10 ng of DNA. DNA amplification was performed in a thermocycler (VWR, Dublin, Ireland) using the recommended parameters. Cleanup and bidirectional sanger sequencing of PCR products was completed by GATC (Eurofins Genomics, Germany).

## 3. Results

All Gram-negative Enterobacterale isolates were phenotypically assessed for β-lactamase and/or *AmpC* genes via selective agars (Figure 2), Double Disk Synergy test (Figure 3), and MIC combination strip method (Figure 4). Genotypic confirmation of β-lactamase and/or *AmpC* genes in all Gram-negative test isolates was determined via PCR.

### 3.1. Characterization of β-Lactamase and AmpC Genes in Gram-Negative Isolates

Out of the three Enterobacterale isolates tested, two (*E. coli* and *K. pneumoniae*) have been confirmed positive for β-lactamase activity by growth on chromatic ESBL agar (Figure 2) and the Double Disk Synergy test (Figure 3). Phenotypic determination of *AmpC* production in *E. coli* and *K. pneumoniae* isolates was further confirmed using MIC combination test strips loaded with cefotetan (CTT)/cefotetan + cloxacillin (CXT) (Figure 4).

**Figure 5:**
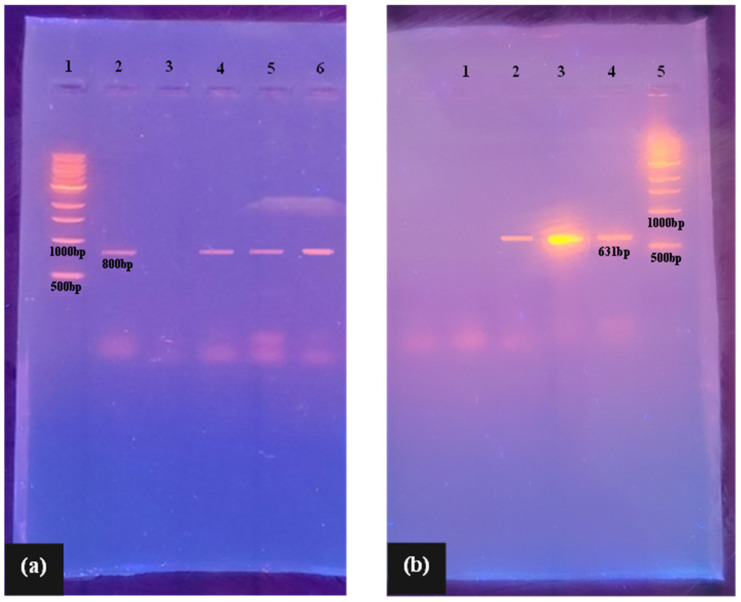
Agarose gel electrophoresis of PCR products of (**a**) ESBL and (**b**) *AmpC*-producing isolates. Image 4 (**a**) Lane 1: 1 kb DNA ladder; Lane 2: quality control strain carrying *bla*_TEM_ gene (positive control); Lane 3: negative control; Lane 4: *bla*_TEM_ positive (*A. baumannii)*; Lane 5: *bla*_TEM_ positive (*K. pneumoniae)*; Lane 6: *bla*_TEM_ positive (*E. coli)*. Image 4 (**b**) Lane 1: negative control; Lane 2: quality control strain carrying *bla*_AmpC_ gene (positive control); Lane 3: *bla*_AmpC_ positive (*E. coli*); Lane 4: *bla*_AmpC_ positive (*P. aeruginosa*); Lane 5: 1 kb DNA ladder.

Molecular methods were used to confirm the presence or absence of β-lactamase (*blaTEM*, *blaSHV*, *blaOXA*, *blaCTX*, and *blaPER*) and/or *AmpC* enzymes (*blaAmpC* and *blaACC)* in Gram-negative test isolates (Figure 5). The results show the WHO critical priority pathogens, *K. pneumoniae*, *E. coli*, and *A. baumannii*, were confirmed positive for β-lactamase activity, with all three isolates harboring the *blaTEM* gene (Figure 5a). *E. coli* was additionally found to concomitantly harbor the *blaAmpC* gene, along with the *P. aeruginosa* isolate (Figure 5b). While notably genotypic data shows the *K. pneumoniae* isolate did not harbour the *AmpC* genotypes (*AmpC*, ACC) tested for in this study, phenotypic results (Figure 4a) suggest the strain may carry other genes belonging to the molecular class C serine β-lactamases (e.g., CIT, EBC, DHA, FOX, or MOX genotypes). Certainly, the susceptibility testing of *K. pneumoniae* further suggests the presence of such genes, where the isolate was resistant to penicillins, narrow-spectrum cephalosporins, third-generation cephalosporins (cefotaxime, ceftazidime, cefixime, and ceftriaxone), and aztreonam. However, it remained susceptible to fourth-generation cephalosporins, including cefepime (MIC of 2 µg/mL) and cefiderocol (MIC of 1.5 µg/mL) (Table 2 and Table 3). This is a pattern that, from a functional standpoint, is typical of *AmpC*-type enzymes. It is also notable that isolates may additionally harbor other β-lactamase genes than those assessed herein, which was limited to TEM, SHV, OXA, CTX, and PER gene types.

### 3.2. Antimicrobial Susceptibility Profile of Isolated Bacterial Zoonotic Pathogens

Table 2 depicts the antimicrobial susceptibility patterns of isolated zoonotic bacterial pathogens, as determined by the Kirby–Bauer disk diffusion assay. Findings show all seven test isolates exhibited a MDR phenotype, presenting with resistance to three or more different antimicrobial classes; every case sampled displayed AMR pathogens in their etiology. Certainly, high levels of MDR were observed among the Gram-positive ESKAPE species, VRE and MRSA, with VRE displaying outright resistance to all tested antibiotic therapy. On the other hand, the MRSA isolate exhibited resistance to streptomycin, penicillins (including the clavulanate inhibitor), third-generation cephalosporins, doxycycline, chloramphenicol [zone of 12 mm falling short of EUCAST epidemiological cutoff value (ECOFF) of 17 mm], and trimethoprim and trimethoprim-sulfamethoxazole (zones of 12 mm falling short of EUCAST cutoff point of 14 mm. The isolate did, however, remain susceptible to macrolide agents (zones ≥ 20 mm), carbapenem agents (zones ≥ 15 mm), and fluoroquinolones (zones ≥ 28 mm), where disk diffusion is unreliable in determining glycopeptide resistance in *Staphylococcus* species (and thus should be reported using MIC methods—see Table 3).

High levels of MDR were further observed among the Gram-negative test species, particularly *E. coli*, *K. pneumonia*, *P. aeruginosa*, and *A. baumannii* isolates. The two Enterobacterale isolates (*E. coli* and *K. pneumonia*) only demonstrated susceptibility to carbapenem agents with zones ≥ 26 mm. *P. aeruginosa* and *A. baumannii* isolates were only susceptible to agents in the carbapenem and fluroquinolone classes, where zones ≥ 15 mm. Notably, all four strains were resistant to third-generation cephalosporins and aztreonam, being attributable to the presence of β-lactamase and/or *AmpC* genes (Figure 5). *A. asburiae* proved to be the most susceptible of the test isolates, being sensitive to streptomycin, cephalosporins, aztreonam, carbapenems, fluoroquinolones, and doxycycline. Albeit the test species did demonstrate resistance to several other antimicrobial agents, including chloramphenicol, penicillins, colistin, trimethoprim, and trimethoprim sulfamethoxazole.

Table 3 shows the MIC results for isolated bacterial species for selected antibiotics and AMPs according to the gradient test strip method, with the MIC method considered the gold standard for determining antimicrobial resistance in species. Results align with the disk diffusion assay, where high levels of MDR were observed among all seven test isolates. Indeed, the antimicrobial-resistant profile of VRE was alarming, where the isolate only demonstrated sensitivity towards one antibiotic agent, dalbavancin. However, with an MIC of 0.5 µg/mL being above the ECOFF of 0.25 µg/mL, the isolate was deemed pan-drug resistant (PDR) or resistant to all test antibiotic therapy. The isolate further demonstrated resistance to the AMP bacitracin. However, it showed susceptibility to the daptomycin AMP with an MIC of 2 µg/mL. MRSA proved resistant to all β-lactam antibiotics, except for the carbapenem agents, where MICs of 3 µg/mL were obtained, respectively, for meropenem and doripenem. MICs obtained for vancomycin (MIC of 3 µg/mL; EUCAST cutoff = 2 µg/mL), dalbavancin (MIC of 1 µg/mL; EUCAST cutoff = 0.125 µg/mL), quinupristin/dalfopristin (MIC of 1.5 µg/mL, EUCAST cutoff = 1 µg/mL), doxycycline (MIC of 16 µg/mL, EUCAST cutoff = 1 µg/mL), and trimethoprim (MIC of 6 µg/mL, EUCAST cutoff = 4 µg/mL), were all above the respective cutoff MICs outlined by EUCAST. Though the MRSA isolate was deemed susceptible to macrolide agents (erythromycin MIC of 1 µg/mL and azithromycin MIC of 2 µg/mL), it is notable that MICs were equivalent to the respective clinical breakpoints outlined by EUCAST. On the other hand, high levels of susceptibility were observed towards ciprofloxacin (MIC of 0.19 µg/mL, EUCAST cutoff 2 µg/mL) gentamicin (MIC of 0.38 µg/mL, EUCAST cutoff 2 µg/mL), and trimethoprim-sulfamethoxazole (MIC of 1 µg/mL, EUCAST cutoff = 4 µg/mL). In addition, the MRSA isolate proved sensitive to both AMPs tested, with MICs of 12 µg/mL and 4 µg/mL produced, respectively, for bacitracin and daptomycin.

The Gram-negative *E. coli* isolate exhibited an XDR profile, where non-susceptibility to at least one agent in all but one or two antimicrobial categories was observed. Indeed, the isolate was only susceptible to one of the fourth-generation cephalosporin, cefiderocol (with a MIC of 0.094 µg/mL), and the carbapenem agents (meropenem and doripenem MICs ≤ 0.047 µg/mL), where MICs all fall within the acceptable EUCAST limits. The activity of β-lactamase inhibitors varied for the *E. coli* species, where clavulanate proved completely ineffective (resistance shown to AMC). However, the addition of tazobactam to piperacillin and avibactam to aztreonam resulted in reduced resistance to these antibiotic agents, where MICs of 3 µg/mL (TZT) and 0.125 µg/mL (AZA) fall within the respective EUCAST MIC breakpoints for both agents. *K. pneumoniae* showed a similar antimicrobial-resistance profile to that of *E. coli*, where the isolate showed resistance to all antimicrobial therapy but fourth-generation cephalosporin (cefepime and cefiderocol MICs ≤ 2 µg/mL), and carbapenem (meropenem and doripenem—MICs ≤ 1 µg/mL) agents. However, unlike the *E. coli* isolate, *K. pneumoniae* further showed resistance to all β-lactamase inhibitors including clavulanate, tazobactam, and avibactam. On the other hand, *K. pneumoniae* was susceptible to another class of antibiotics, the aminoglycosides, where an MIC of 1.5 µg/mL for gentamicin falls within the acceptable EUCAST limit of 2 µg/mL. The opportunistic environmental isolates, *P. aeruginosa* and *A. baumannii*, additionally demonstrated high levels of resistance to a myriad of antimicrobial classes, with the exception of carbapenems (MICs ≤ 1.5 µg/mL) and fluoroquinolones (MICs ≤ 0.5 µg/mL). *P. aeruginosa* further showed sensitivity towards fourth-generation cephalosporins (MICs ≤ 4 µg/mL), while *A. baumannii* exhibited susceptibility to gentamicin (MIC 0.25 µg/mL). The preference towards β-lactamase inhibitors for these species varied, where clavulanate was ineffective against both isolates when combined with amoxicillin. Tazobactam was more effective against *P. aeruginosa* (MIC of 16 µg/mL for TZT) than *A. baumannii* (MIC of 64 µg/mL for TZT) when combined with piperacillin, where the EUCAST breakpoint for penicillin agents for these species is 16 µg/mL. Likewise, the addition of avibactam to aztreonam proved more effective against *P. aeruginosa* (3 µg/mL µg/mL) than *A. baumannii* (64 µg/mL), where the defined EUCAST breakpoint for aztreonam for these species is 16 µg/mL. Notably, the high level of resistance towards β-lactam agents for *E. coli*, *K. pneumoniae*, *P. aeruginosa*, and *A. baumannii* was owed to the presence of ESBL enzymes and/or AmpC type-enzymes (Figure 5).

Although *E. asburiae* proved to be the most sensitive of the isolates to antibiotic therapy, the species was deemed to exhibit a MDR phenotype, being resistant to macrolides, penicillins, cefixime, gentamycin (MIC of 3 µg/mL, EUCAST cutoff = 2 µg/mL), doxycycline (MIC of 4 µg/mL, EUCAST cutoff = 0.5 µg/mL), colistin (MIC of 4 µg/mL, EUCAST cutoff = 2 µg/mL), chloramphenicol (MIC of 8 µg/mL, EUCAST cutoff = 4 µg/mL), and trimethoprim. On the other hand, the isolate showed high levels of susceptibility towards extended spectrum cephalosporin agents (except for cefixime), carbapenems, monobactams, fluoroquinolones, and trimethoprim-sulfamethoxazole, where MICs for all agents were ≤ 0.25µg/mL. Moreover, the isolate was the only Gram-negative species to phenotypically test negative for the presence of ESBL genes, where susceptibility testing indicates the isolate likely harbours broad-spectrum β-lactamase enzymes (where resistance to penicillins, narrow-spectrum cephalosporins, and cefixime was observed). This is further corroborated by the isolate being susceptible to all β-lactamase inhibitors, where *E. asburiae* was the only Gram-negative isolate to be deemed susceptible to AMC (MIC of 3 µg/mL), where clavulanate is known to be more active against broad spectrum β-lactamase producers, rarely being active against ESBL-producing strains. Furthermore, all five Gram-negative isolates demonstrated outright resistance to both AMPs (BAC and DAP) tested in this study, where treatment options are becoming limited in the management of Gram-negative pathogens.

### 3.3. Antimicrobial-Susceptibility Profile of Isolated Fungal Zoonotic Pathogens

Table 4 displays evident resistance to fluconazole in many *Candida* species including *C. albicans*, *glabrata*, and *krusei* with susceptibility present in *C. parapsilosis* and *C. neoformans* and resistance to flucytosine in all species tested. AMP B demonstrated toxicity to all species except *C. krusei* at 20 µg. At 100 µg fluconazole provided significant levels of activity against *C. parapsilosis* and *C. neoformans*.

Table 5 displays MIC concentrations for test species with AMP B again proving most effective with *C. parapsilosis* not achieving any MIC value. No MIC value was obtained for flucytosine or both AMPs tested for any fungal species investigated. Caspofungin demonstrated activity against *C. albicans* and *C. glabrata* with fluconazole providing an MIC of 3 and 6 µg/mL against *C. parapsilosis* and *C. neoformans*, respectively.

## 4. Discussion

Studies described herein demonstrate clear MDR and XDR in many clinically relevant pathogens of zoonotic origin. All WHO critically and high-priority bacterial and fungal species tested possess resistance to a broad range of therapeutic options with resistance genes present. *E. coli* displayed XDR resistance, limiting the therapeutic options available for this strain [13]. MDR *E. coli* is associated with high mortality rates, especially in immunocompromised persons, including neonates. Indeed, sepsis resultant from *E. coli* bacteraemia has a mortality rate of 33% after 30 days [9]. Studies report bacteraemia associated with *E. coli* is resistant to aminoglycosides, cephalosporins, penicillin, fluoroquinolones, and B-lactam combination agents [14], with similar findings to this study. The presence of β-lactamase activity in *E. coli* and *K. pneumoniae* isolates further confirms current research indicating that the incidence of ESBL-producing *Escherichia coli* and *Klebsiella* spp. strains are increasing [15]. EBSL-producing Enterobacteroles generally remain susceptible to carbapenems; ESBL enzymes do not inactivate non-β-lactam agents (e.g., ciprofloxacin, trimethoprim-sulfamethoxazole, gentamicin) [16]. ESBL *E. coli* described here displays resistance to ciprofloxacin, gentamicin, and trimethoprim-sulfamethoxazole, with only gentamicin having efficacy against ESBL *K. pneumonia*. Piperacillin-tazobactam achieved satisfactory MICs against Gram-negative strains except *E. coli* and *E. asburiae*. Furthermore, hypervirulent strains of *K. pneumoniae* have emerged possessing extra virulence factors displaying resistance to cephalosporin, cephamycin, monobactam, aminoglycosides, fluoroquinolone, and trimethoprim-sulfamethoxazole [17]. The *K. pneumonia* isolate tested only displayed sensitivity to the carbapenems and may prove to be a hypervirulent strain. Notably, both *E. coli* and *K. pneumoniae* tested are resistant to third-generation cephalosporins, where Enterobacterales exhibiting third-generation cephalosporin-resistance are classified as critical priority by the WHO. The high rate of resistance towards these agents was attributable to the presence of β-lactamase genes, where both species were found to harbor the *bla*_TEM_ gene. In addition, both isolates tested phenotypically positive for AmpC-type-enzymes, where *E. coli* was found to carry the *bla*_AmpC_ gene. The presence of AmpC enzymes in isolates can further confer resistance to beta lactamase inhibitors, where resistance to amoxicillin clavulanate was observed for all Gram-negatives tested. *P. aeruginosa* and *A. baumannii* also possess ESBL enzymes and/or *AmpC*-type-enzymes conferring resistance on both species, which prove MDR. Clinically, the potent MDR profile and virulence of *P. aeruginosa* results in alarming mortality rates of ca. 56% [18].

*E. asburiae* proved to be the most sensitive of the isolates to antibiotic therapy. The species, however, was deemed to be an MDR phenotype, being resistant to macrolides, penicillins, cefixime, and gentamycin. This is an important find as *E. asburiae*, which belongs to the *Enterobacter cloacae* complex (ECC) group, is emerging clinically in the aetiology of pneumonia, urinary tract infections (UTIs), and septicemia [19]. Pathogens having ESBL genes often possess additional genes or gene mutations that mediate resistance to a broad range of antibiotics, conferring XDR [16]. VRE displays pan drug resistance (PDR), having resistance to all antimicrobial agents tested where dalbavancin failed to achieve the ECOFF value for VRE. Dalbavancin, a lipoglycopeptide antibiotic used to treat MRSA dermal infections, failed to achieve the required MIC for MRSA in this study (i.e., 0.125 µg/mL). The Infectious Diseases Society of America (IDSA) guidelines recommend that MRSA bacteraemia be treated with vancomycin if the MIC is <2 μg/mL, based on CLSI breakpoints [20]. The MIC for vancomycin against MRSA in this study was 3 μg/mL, hindering the application of vancomycin against this strain. Studies report that MRSA bloodstream infections (BSIs) having a vancomycin MIC of >1 μg/mL are associated with increased treatment failure and patient mortality [21]. Importantly, the AMPs bacitracin and daptomycin did not provide significant inhibition of bacterial species tested. Bacitracin is a broad-spectrum AMP produced by *Bacillus subtilis* and *Bacillus licheniformis* traditionally displaying an inhibitory effect against Gram-positive bacteria *S. aureus*, *Streptococcus*, and *Enterococcus* [22]. VRE tested in this study displayed resistance to BAC with an MIC of 12 µg/mL obtained for MRSA. The cyclic lipopeptide daptomycin is produced by *Streptomyces roseosporus* and is currently listed by the WHO as a last-resort antibiotic for the treatment of Gram-positive infections [11]. Daptomycin resistance is associated with altered membrane composition in both *S. aureus* and *B. subtillis* species. MICs of 2 and 4 µg/mL were achieved for VRE and MRSA, respectively, in this study. The MIC for MRSA is significantly higher than that obtained in the study of Diederen et al., (2006) where an MIC range for daptomycin of 0.125 to 1.0 μg/mL was reported [23]. Daptomycin was approved for clinical use in 2003 with no new classes of antibiotics approved since to treat the emerging threat of ESKAPE pathogens.

All fungals isolated investigated displayed high levels of resistance and MDR. EUCAST breakpoints are not currently available for caspofungin, due to significant inter-laboratory variation in MIC values. Caspofungin only displayed efficacy against *C. albicans* and *C. glabrata* in this study. *Candida* species are causative of high rates of fungal BSI ca. 93% and late-onset sepsis in neonates with a ca. 70% mortality rate [24]. Low- and middle-income countries have increasing prevalence of fluconazole-resistant isolates, including *C. parapsilosis*, *C. krusei*, and *C. auris* [25]. The prophylactic use of fluconazole clinically has proliferated the emergence of non-albicans *Candida* strains, an alarming trend as fluconazole is better tolerated by infant patients [26]. The MIC of azoles against *C. glabrata* and *C. krusei* is higher due to the intrinsic resistance in these species with *C. glabrata* acquiring resistance to echinocandins, being recommended for the treatment of candidemia [27]. Meningitis associated with *Cryptococcus* species has a high mortality rate of 78% and 42% in HIV-positive and negative patients, respectively [28]. *C. neoformans* possesses intrinsic resistance to azole antifungals, requiring IFIs with this species to be treated with 5-fluorocytosine combined with AMP B [29]. The strain investigated in this study, however, displayed resistance to 5-fluorocytosine up to 32 µg/mL. Resistance to 5-fluorocytosine is achieved by preventing cellular uptake or enzymatic changes converting 5FC to active 5-FU. AMP B and fluconazole limit this resistance by disrupting the fungal cell wall integrity, allowing for 5FC uptake into the cell [30]. Fungal metaphylaxis is difficult due to intrinsic and acquired MDR and drug biocompatibility issues in vivo. AMP B proved to be the only effective antifungal tested against all isolates in this study (except *C. krusei)* as determined via zone of inhibition but failed to achieve the desired levels of inhibition via MIC assay. AMP B is used as a stand-alone therapy or given in conjunction with flucytosine for systemic fungal infections. AMP B is associated with many organ toxicities, including kidney, liver, and blood toxicity. Flucytosine is associated with many side effects, including bone marrow depression, neutropenia, hepatotoxicity, diarrhea, and vomiting with prolonged elevated blood flucytosine concentrations of >100 mg/L. Furthermore, flucytosine has teratogenic activity rat species [31]. Horizontal gene transfer (HGT) of resistance genes is a factor proliferating AMR amongst microbial species. AMR genes present in the environment are a risk to ecosystem biodiversity and the transmission of AMR to wildlife, domestic animals, and humans, zoonotic AMR [32]. As shown in this study, animals act as reservoirs of AMR, which may transmit via food, water, and agricultural practices, causing zoonotic difficult-to-treat disease. The One Health approach aims to improve antimicrobial use and introduce regulation and policy to reduce animal and human infectious disease and the associated AMR. To this end, effective surveillance, antimicrobial stewardship, infection control, sanitation, animal husbandry, and alternatives to antimicrobials are needed. The findings of this study support the research of Jin et al. (2023), where species including MRSA, ESBL/*AmpC*-producing Enterobacterales and MDR Gram-negative species can be transmitted zoonotically [33]. Studies described herein also support the research of Woerde et al. (2023), identifying ESBL-resistance genes in *E. coli* and *K. pneumoniae*, *Enterobacter* spp., and *Pseudomonas aeruginosa* [33]. Further studies are also warranted to determine the rate and impact of anthropozoonosis from a One Health perspective, where owners may spread pathogens to animal companions, which act as reservoirs.

## 5. Conclusions

Studies described herein demonstrate the alarming rate of resistance in critically important species isolated from companion animals displaying morbidity. All ESKAPE and fungal pathogens investigated possess MDR or greater resistance profiles making them difficult to treat. In an era of antimicrobial resistance, without novel therapeutic options the rate of mortality from infectious disease will increase to ca. 10 million in 2050. Healthcare costs and mortality rates for patients are also significantly higher than those with infections involving drug-susceptible pathogens.

Undoubtedly, animals are a reservoir facilitating transmission of antibiotic-resistant pathogens to humans, other animals, and the natural environment. Importantly, the One Health methodology aims to reduce this interconnected route of transmission, a difficult task as animals become more commonplace in society. Therapy animals, for example, have proven beneficial in the recovery of patients in clinical settings. Therapy dog programs in hospitals, aged care facilities, education centers, and mental health facilities have been reported to enhance socialization and reduce depression and/or anxiety in patients. Environmental contamination with AMR genes such as those identified in this study and horizontal gene transfer enables the emergence of other MDR species. These pathogens severely limit treatment options, increase and prolong morbidity, and increase mortality rates. This is a critical issue and global public health emergency requiring a multifaceted approach with improved surveillance, improved point of care diagnostics, effective antimicrobial stewardship, and increased prevention strategies. To improve antimicrobial stewardship, effective screening of pathogens for AMR profiling will allow for optimal treatment protocols. Future studies assessing the relationship between such zoonotic species, AMR profiling, and LPS secretion may inform on zoonotic sepsis risk, where sepsis remains a difficult-to-treat condition having high mortality rates. Additional studies are required at molecular and genetic levels to elucidate the mechanisms of AMR propagation in zoonotic species.

## Figures and Tables

**Table 1:** Details of primers used to identify bacterial and fungal isolates in this study. ESBL oligonucleotide primers used are also listed.

Target Gene	Primer Name	Primer Sequence	Expected Amplicon Size
16 s rRNA	ITS_8F	AGGTTTGATCCTGGCTCAG	1500 bp
	ITS_U1492R	GGTTACCTTGTTACGACTT	
ITS	ITS1-F	CTTGGTCATTTAGAGGAAGTAA	600 bp
	ITS4	TCCTCCGCTTATTGATATGC	
blaTEM	TEM-F	CATTTCCGTGTCGCCCTTATTC	800 bp
	TEM-R	CGTTCATCCATAGTTGCCTGAC	
blaSHV	SHV-F	AGCCGCTTGAGCAAATTAAAC	712 bp
	SHV-R	ATCCCGCAGATAAATCACCAC	
blaCTX-M group 1	CTXM1-F	TTAGGAAATGTGCCGCTGTA	688 bp
	CTXM1-R	CGATATCGTTGGTGGTACCAT	
blaCTX-M group 2	CTXM2-F	CGTTAACGGCACGATGAC	404 bp
	CTXM2-R	CGATATCGTTGGTGGTACCAT	
blaAmpC	AMPC-F	CCCCGCTTATAGAGCAACAA	631 bp
	AMPC-R	TCAATGGTCGACTTCACACC	

**Table 2:** Zones of inhibition (mm) produced by test species to a range of antibiotics from varying drug classes with EUCAST target zones to indicate susceptibility represented by alphabetical lettering.

Drug Class		AG	G	C	Macrolide		Penicillin’s		Cephalosporins			MB	Carbapenems			Quinolones		PM	TM	SXT	TET
Antibiotic		S	VA	C	E	AZM	AMP	AMC	CPD	CTX	CRO	ATM	MEM	DOR	IMP	LEV	CIP	CS	TM	SXT	DO
Conc. (µg/disc)		10	30	30	15	15	10	20:10	10	5	30	30	10	10	10	5	5	10	5	25	10
Bacterial Isolate	VRE	R	R ^A^	-	R	R	R	R	-	-	-	-	R	R	R	R ^B^	R ^B^	-	R	R	R
	MRSA	R	17	12	22 ^c^	20	R ^D^	10	R	R	R	-	16	15	27	30 ^E^	28 ^f^	-	12 ^G^	12 ^G^	R ^f^
	*K. pneumoniae*	R	-	R ^E^	R	R	R	R ^H^	9	12 ^E^	13 ^f^	R ^c^	32 ^I^	32 ^c^	23 ^f^	R ^H^	R ^f^	11	R ^B^	R ^L^	R
	*A. Baumannii*	13	-	R	R	R	R	R	R	R	R	R	20 ^B^	15 ^f^	27	20 ^J^	17 ^c^	R	R	R	12
	*P. aeruginosa*	9	-	R	R	12	R	R	R	R	R	10 ^K^	22 ^G^	23 ^f^	25	20 ^K^	22 ^D^	10	R	R	R
	*E. asburiae*	17	-	15 ^E^	R	12	R	12 ^H^	26	29 ^E^	29	35 ^c^	27 ^I^	18 ^c^	26 ^f^	30 ^H^	32 ^f^	12	12 ^B^	17 ^L^	18
	*E. coli*	11	-	R ^E^	R	11	R	R ^H^	R	R ^E^	R	14 ^c^	26^I^	25 ^c^	28 ^f^	9 ^H^	14 ^f^	10	R ^B^	R ^L^	9

(A = 12 mm, B = 15 mm, C = 21 mm, D = 26 mm, E = 17 mm, F = 22 mm, G = 14 mm, H = 19 mm, I = 16 mm, J = 20 mm, K = 18 mm, L = 11 mm) where S denotes “Susceptible”, I denotes “Susceptible, increased exposure”, and R denotes “Resistance” to antibiotic drugs. Abbreviations: AG—Aminoglycoside, S—Streptomycin, G—Glycopeptide, VA—Vancomycin, C-Chloramphenicol, E—Erythromycin, AZM—Azithromycin, AMP—Ampicillin, AMC—Amoxicillin clavulanate, CPD—Cefpodoxime, CTX—Cefotaxime, CRO—Ceftriaxone, MB—Monobactam, ATM—Aztreonam, MEM—Meropenem, DOR—Doripenem, IMP—Imipenem, LEV—Levofloxacin, CIP—Ciprofloxacin, PM—Polymyxin, CS—Colistin, TET-Tetracycline, TM—Trimethoprim, SXT—Trimethoprim-sulfamethoxazole, DO—Doxycycline.

**Table 3:** MIC results for isolated bacterial species for selected antibiotics and AMPs according to the gradient test strip method. EUCAST MIC cutoff points for susceptibility are represented by alphabetical lettering (A = 4 µg/mL, B = 1 µg/mL, C = 8 µg/mL, D = 128 µg/mL, E = 2 µg/mL, F = 0.125 µg/mL, G = 0.5 µg/mL, and H = 16 µg/mL), where S denotes “Susceptible”, I denotes “Susceptible, increased exposure”, and R denotes “Resistance” to antibiotic drugs.

Antimicrobial		Bacterial Isolate						
		VRE	MRSA	*K. pneumoniae*	*A. Baumannii*	*P. aeruginosa*	*E. asburiae*	*E. coli*
Agent	Strip Range (µg/mL)	MIC (µg/mL)						
Macrolides								
Erythromycin (E)	0.016–256	R	1 ^B^	R	R	R	R	R
Azithromycin (AZM)	0.016–256	R	2 ^E^	R	R	R	R	48
Glycopeptide								
Vancomycin (VA)	0.016–256	R ^A^	3 ^E^	-	-	-	-	-
Dalbavancin (DAL)	0.002–32	0.5	1 ^f^	-	-	-	-	-
Streptogramin								
Quinupristin/dalfopristin (QDA)	0.002–32	R	1.5 ^B^	-	-	-	-	-
Penicillins								
Amoxicillin (AML)	0.016–256	R ^c^	R	R ^c^	R	R	R ^c^	R ^c^
Amoxicillin-clavulanic acid (2 µg/mL) (AMC)	0.016/2–256/2	R ^c^	8	R ^c^	R	R	3 ^c^	16 ^c^
Piperacillin (PIP)	0.016–256	R	R	R ^c^	R	R ^H^	R ^c^	R ^c^
Piperacillin-tazobactam (4 µg/mL) (TZP)	0.016/4–256/4	R	R	16 ^c^	64	16 ^H^	2 ^c^	3 ^c^
Cephalosporins								
Cefixime (CFM)	0.016–256	-	R	16 ^B^	16	R	R ^B^	R ^B^
Ceftazidime (CAZ)	0.016–256	-	128	8 ^A^	R	12 ^c^	0.047 ^A^	6 ^A^
Ceftriaxone (CRO)	0.016–256	-	128	8 ^E^	R	R	0.125	R ^E^
Cefepime (FEP)	0.002–32	-	96	2 ^A^	R	4 ^c^	0.047	R ^A^
Cefiderocol (FDC)	0.016–256	-	R	1.5 ^E^	R	2 ^E^	0.016	0.094 ^E^
Carbapenems								
Doripenem (DOR)	0.002–32	R	3	0.094 ^E^	1.5 ^E^	1 ^E^	0.125 ^E^	0.012 ^E^
Meropenem (MEM)	0.002–32	R	3	1 ^c^	0.25 ^A^	0.19 ^c^	0.064 ^c^	0.047 ^c^
Monobactams								
Aztreonam (ATM)	0.016–256	-	-	R ^A^	R	24 ^H^	0.19 ^A^	8 ^A^
Aztreonam-Avibactam (AZA)	0.016/4–256/4	-	-	R	64	3	0.023	0.125
Fluoroquinolones								
Ciprofloxacin (CIP)	0.002–32	R ^A^	0.19 ^E^	R ^G^	0.5 ^B^	0.125 ^G^	0.023 ^G^	3 ^G^
Aminoglycosides								
Gentamicin (CN)	0.016–256	R ^D^	0.38 ^E^	1.5 ^E^	0.25 ^A^	3	3 ^E^	R ^E^
Tetracyclines								
Doxycycline (DXT)	0.016–256	R	16 ^B^	64 ^G^	2	R	4 ^G^	24 ^G^
Miscellaneous Agents								
Colistin (CS)	0.016–256	-	-	8 ^E^	32 ^E^	6 ^A^	4 ^E^	4 ^E^
Chloramphenicol (C)	0.016–256	-	48	R	R	R	8 ^A^	R ^A^
Trimethoprim (TM)	0.002–32	R	6 ^A^	R ^A^	R	R	R ^A^	R ^A^
Trimethoprim-sulfamethoxazole (1:19) (SXT)	0.002–32	R	1 ^A^	R ^A^	R ^A^	R	0.25	R
Antimicrobial Peptides								
Bacitracin (BA)	0.016–256	R	12	R	R	R	R	R
Daptomycin (DAP)	0.016–256	2	4	R	R	R	R	R

**Table 4:** Zones of inhibition (mm) produced by fungal priority pathogens for various antifungal test agents.

Drug Class.		Polyenes	Azoles	Antimetabolite
Antifungal		AMP B	Fluconazole	Flucytosine
Concentration (µg/disc)		20	100	1
Fungal Isolate	*C. albicans*	15	R	R
	*C. glabrata*	10	R	R
	*C. krusei*	R	R	R
	*C. parapsilosis*	13	30	R
	*C. neoformans*	14	30	R

**Table 5:** MIC results for isolated fungal species for selected antifungal agents and AMPs according to the gradient test strip method. EUCAST target zones to indicate susceptibility is represented by alphabetical lettering (A = 1 µg/mL, B = 4 µg/mL, C = 16 µg/mL) where S denotes “Susceptible”, I denotes “Susceptible, increased exposure”, and R denotes “Resistance” to antifungal drugs.

Antifungal		*C. albicans*	*C. glabrata*	*C. krusei*	*C. parapsilosis*	*C. neoformans*
Test Agent	Strip Range MIC (µg/mL)	MIC (µg/mL)				
Polyenes						
AMP B	0.002–32	0.19 ^A^	0.125 ^A^	0.19 ^A^	R ^A^	0.75 ^A^
Azoles						
Fluconazole	0.016–256	R ^B^	R ^c^	-	3 ^B^	6 ^B^
Echinocandins						
Caspofungin	0.002–32	0.38	0.5	R	R	-
Antimetabolite						
Flucytosine	0.002–32	R	R	R	R	R
Antimicrobial Peptides						
Bacitracin	0.016–256	R	R	R	R	R
Daptomycin	0.016–256	R	R	R	R	R

Note: Until breakpoints are established for caspofungin by EUCAST, MIC breakpoints can be extrapolated from anidulafungin (4 µg/mL) and micafungin (2 µg/mL) for *C. parapsilosis*.

## Data Availability

The raw data supporting the conclusions of this article will be made available by the authors on request.
